# Hybrid assembly using long reads resolves repeats and completes the genome sequence of a laboratory strain of *Staphylococcus aureus* subsp. aureus RN4220

**DOI:** 10.1016/j.heliyon.2022.e11376

**Published:** 2022-11-02

**Authors:** Suresh Panthee, Hiroshi Hamamoto, Atmika Paudel, Chikara Kaito, Yutaka Suzuki, Kazuhisa Sekimizu

**Affiliations:** a Drug Discoveries By Silkworm Models, Faculty of Pharma-Science, Teikyo University, Tokyo, Japan; b GenEndeavor LLC, 26219 Eden Landing Rd, Hayward, CA, 94545, USA; c Teikyo University Institute of Medical Mycology, 359 Otsuka, Hachioji, Tokyo, 192-0395, Japan; d Division of Infection and Immunity, International Institute for Zoonosis Control, Hokkaido University, North 20, West 10, Kita-ku, Sapporo, Hokkaido 001-0020, Japan; e Graduate School of Medicine Dentistry and Pharmaceutical Sciences, Okayama University, Okayama, Japan; f Department of Medical Genome Sciences, Graduate School of Frontier Sciences, The University of Tokyo, Chiba, Japan

**Keywords:** *Staphylococcus aureus*, RN4220, Complete genome, Hybrid assembly, RNA-Seq, Repeat regions, Staphyloxanthin

## Abstract

*Staphylococcus aureus* RN4220 has been extensively used by staphylococcal researchers as an intermediate strain for genetic manipulation due to its ability to accept foreign DNA. Despite its wide use in laboratories, its complete genome is not available. In this study, we used a hybrid genome assembly approach using minION long reads and Illumina short reads to sequence the complete genome of *S. aureus* RN4220. The comparative analysis of the annotated complete genome showed the presence of 39 genes fragmented in the previous assembly, many of which were located near the repeat regions. Using RNA-Seq reads, we showed that a higher number of reads could be mapped to the complete genome than the draft genome and the gene expression profile obtained using the complete genome also differs from that obtained from the draft genome. Furthermore, by comparative transcriptomic analysis, we showed the correlation between expression levels of staphyloxanthin biosynthetic genes and the production of yellow pigment. This study highlighted the importance of long reads in completing microbial genomes, especially those possessing repetitive elements.

## Highlights

• *S. aureus* RN4220 is used as an intermediate strain for genetic manipulation.
• We completed its genome and found 39 fragmented genes in previous genome assembly.
• RNA-Seq analysis improved mapping of the reads with the use of complete genome.
• Expression of staphyloxanthin biosynthetic genes was correlated with its production.

## 1 Introduction

*Staphylococcus aureus* is a Gram-positive bacterium capable of opportunistic infections, which can sometimes be fatal. Genetic manipulation of *S. aureus* was limited until *S. aureus* strain RN4220 was obtained by chemical mutagenesis of *S. aureus* NCTC8325-4 strain [1]. NCTC8325-4 is a derivative of a clinical isolate NCTC8325 obtained by curing the three prophages Φ11, Φ12, and Φ13 [2]. Therefore, both NCTC8325-4 and RN4220 lack the three prophages. In addition, RN4220 can accept foreign DNA and is characterized by a mutation in the *sauI hsdR* gene belonging to the restriction-modification system [3]. Due to this property, RN4220 is routinely used in the laboratories as an intermediate for genetic manipulation; plasmids from *Escherichia coli* are electro-transformed into RN4220, and the plasmids from RN4220 are then transformed to another *S. aureus* strain by suitable methods such as phage transduction.

Despite its wide use, the complete genome of this strain is not available. With the recent development in next-generation sequencing technologies, there have been attempts to sequence the genome. Apart from our assembly, there are two deposited assemblies of RN4220 in NCBI. The first assembly was done in 2011 using Illumina GA II [4] (accession: GCA_000212435.2), and the second was performed in 2020 using BGISeq (accession: GCA_011751615.1), which generated 118 and 27 contigs, respectively. Whereas the assemblies primarily provided valuable information regarding the genetic make-up of this strain, we still need a complete genome sequence to make the most out of this laboratory strain. Short reads sequencing of the genome can be attributed to the large number of contigs generated from these assemblies. Short-read assemblies are challenged by the presence of identical sequences at more than one locus of the chromosome called the repetitive DNA sequences, or repetitive elements or repeated regions, based on which the microbial genomes can be categorized into three classes with varying degrees of difficulty in genome assembly [5].

Larger organisms such as eukaryotes have many repetitive elements throughout the genome; for instance, nearly half of the human genome consists of repetitive elements [6]. Bacteria such as *Orientia tsutsugamushi* possess 37% repetitive elements throughout their genome [7] and analysis of roughly 10,000 complete bacterial/archaeal genomes indicated that up to 10% of prokaryotic genomes could be either very repeat-rich and/or harbor long repeats, both of which require long-read sequence data to fully resolve their genome sequences [8]. Significant attention has been paid to *S. aureus* due to its pathogenic ability. By the end of 2021, more than 26000 genome assemblies for *S. aureus* are available at NCBI; however, little more than 1000 have been completed. Although there might be several reasons behind this lag, it can partly be attributed to short-read sequencers and the presence of repeat regions. A well understandable example of a repeat region includes 16s rRNA, located at six different positions in the genome. In this study, by hybrid assembly using both long and short reads, we completed the genome of RN4220. Upon further analysis, we found many repetitive elements and several fragmented genes in the previous assembly. Our approach using long reads was suitable for covering those repetitive regions, and we were successful in obtaining a complete genome.

## 2 Materials and methods

### 2.1 Genome sequencing, assembly, and annotation

*S. aureus* RN4220 was routinely cultured on tryptic soy broth at 37 °C without antibiotic selection. Genome sequencing and assembly were performed as previously explained [9, 10, 11, 12]. Briefly, genomic DNA was isolated from overnight culture using Qiagen DNA-blood Mini Kit (Qiagen, Hilden, Germany) and lysostaphin for bacterial lysis. Construction of short-read single-end libraries was performed using Illumina TruSeq DNA Sample Preparation Kit (Illumina, San Diego, CA, USA) [13]. After confirming the quality and quantity of the constructed libraries, subsequent sequencing was performed using the Illumina HiSeq2000. Quality filtering and adapter trimming of the reads were performed using CLC Genomics workbench. MinION long reads sequencing was performed using 1 μg genomic DNA. Hybrid error correction of the long reads was performed by LoRDEC [14] using the short reads, and the final assembly of the circular chromosome was performed using Flye 2.3.3 [15]. Short reads were then mapped to the chromosome, and the consensus was generated to obtain the final assembly. The final assembly was then annotated using the NCBI Prokaryotic Genome Annotation Pipeline (PGAP) [16].

### 2.2 Comparative genomic analysis

The complete genome sequence of the parent strain NCTC8325 and two draft assemblies of the RN4220 strain were downloaded from NCBI. The draft assemblies were first aligned using Mauve Contig Mover [17]. The ordered contigs were then submitted to the CLC Genomics workbench for whole-genome alignment. The annotations were checked manually to identify the genes fragmented in the previous assembly. Repeat finding of the genome was performed using Unipro UGENE v.39.0 [18]. To analyze the gene gain and loss events in RN4220, we used five strains- NCTC8325- the parent strain, NBRC100910^T^-the type strain, and other virulent strains- Smith, Newman, and JE2. The genomes were first analyzed by M1CR0B1AL1Z3R [19] to generate the whole-genome phylogeny and phyletic patterns. The outputs were then submitted to GLOOME [20] to map gene gain and loss using default parameters.

### 2.3 Staphyloxanthin biosynthesis pathway analysis

Raw RNA-Seq reads for *S. aureus* RN4220, NCTC8325, and SH1000 were downloaded from NCBI SRA. The SRA accession numbers and BioProject details are summarized in Table 1. The reads were then mapped to their respective complete genome using the CLC Genomic Workbench ver 20.0.4 (CLC bio, Aarhus, Denmark). Since the complete genome sequence of the SH1000 strain is not publicly available, the SH1000 reads were mapped to the NCTC8325 genome. Transcripts Per Million (TPM), a sequence depth normalized indicator for expression analysis, was used to compare the expression level among the study samples.

**Table 1:** SRA accession numbers used in this study.

*S. aureus*	BioProject	Sample	SRA accession	Sequencing method	Institute
RN4220	PRJDB5479	1_RN4220-1	DRR084259	Illumina HiSeq 2500	University of Tokyo Graduate School of Pharmaceutical Sciences
		2_RN4220-2	DRR084260		
		3_RN4220-3	DRR084261		
		4_DcvfE-1	DRR084262		
		5_DcvfE-2	DRR084263		
		6_DcvfE-3	DRR084264		
NCTC8325	PRJNA433003 [21]	wt1	SRR6674886	Illumina HiSeq 2500	University of British Columbia
		wt2	SRR6674887		
		wt3	SRR6674888		
SH1000	PRJNA682641 [22]	PL_TSB_1	SRR13200515	Illumina NextSeq 500	Université de Sherbrooke
		PL_TSB_2	SRR13200516		
		PL_TSB_3	SRR13200517		
		Bio_TSB_1	SRR13200521		
		Bio_TSB_2	SRR13200522		
		Bio_TSB_3	SRR13200523		
		PL_BHI_1	SRR13200518		
		PL_BHI_2	SRR13200519		
		PL_BHI_3	SRR13200520		
		Bio_BHI_1	SRR13200524		
		Bio_BHI_2	SRR13200525		
		Bio_BHI_3	SRR13200526		
	PRJNA472336 [23]	WT1_S7	SRR7189482	Illumina NextSeq 500	University of Leeds, UK
		WT3_S9	SRR7189483		

## 3 Results and discussions

### 3.1 Completion of the genome sequence of *S. aureus* RN4220

**Figure 1:**
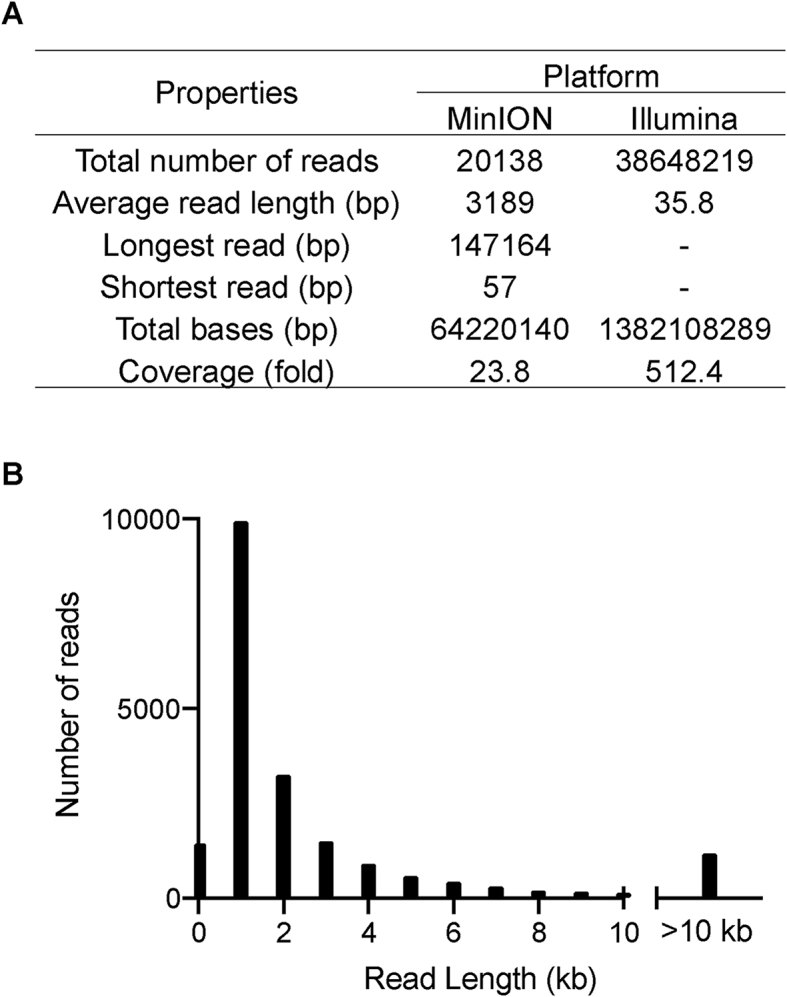
Genome sequencing of *S. aureus* RN4220 using hybrid-genome assembly approach. (A) Summary of sequence reads from MinION and Illumina sequencers. (B) Histogram of read length obtained from the MinION sequencer.

Previous attempts to sequence the RN4220 genome used sequencing platforms that produced short reads. *S. aureus* NCTC8325, the parent strain of RN4220, also possesses repetitive elements, which create a challenge in assembling the genome using short reads. To cover the repetitive elements while sequencing a genome, either the reads longer than the repetitive elements or alternative approaches to overcoming this problem are essential. In this study, we took advantage of our hybrid genome assembly approach [9, 10, 11, 12] using long reads from ONT MinION and short reads from Illumina to complete the RN4220 genome. As high-quality long reads are much more crucial than coverage, we could complete the genome of this bacterium with low coverage long reads and high coverage short reads. The summary of reads obtained from the MinION and Illumina platforms are shown in Figure 1A, B.

**Figure 2:**
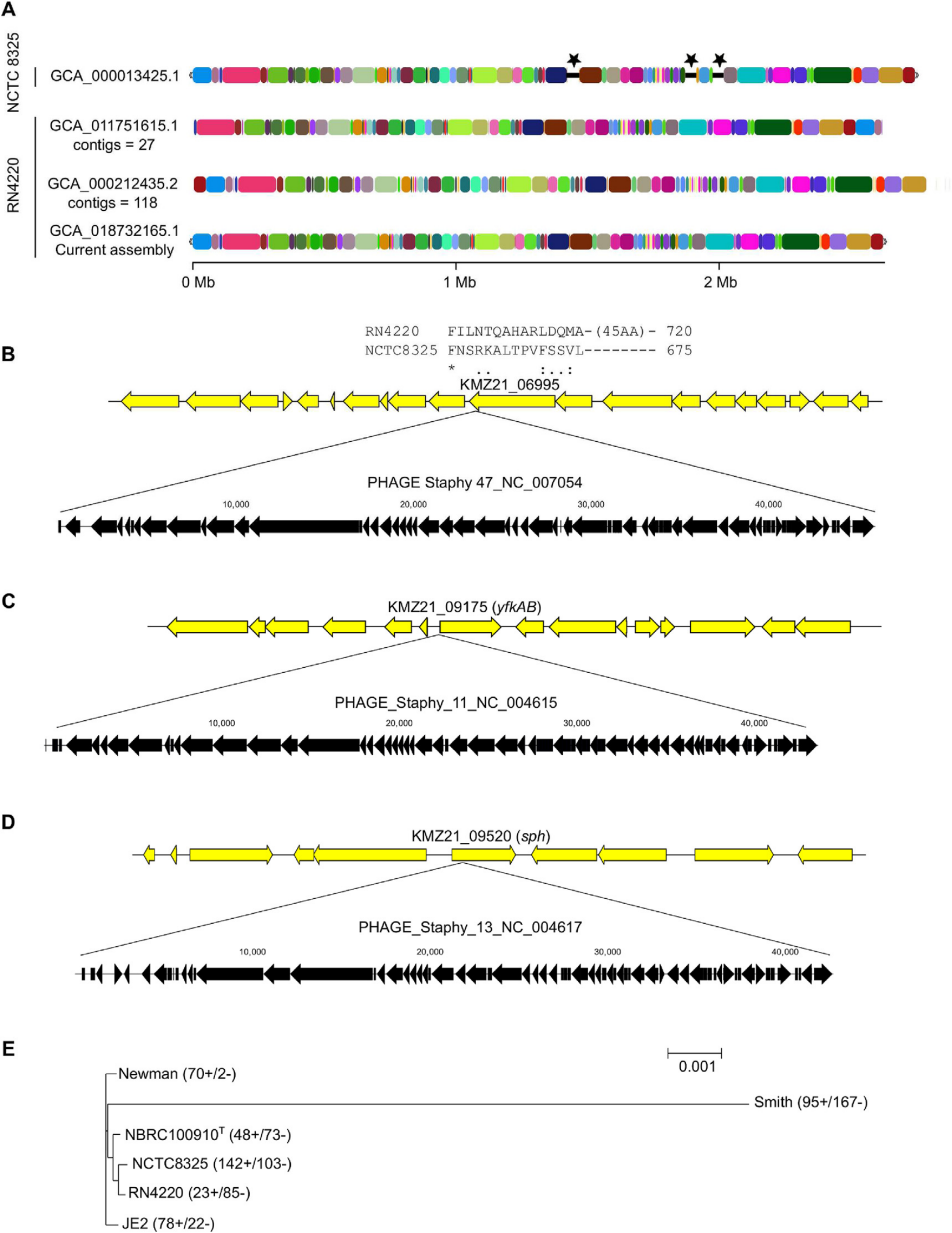
Comparative analysis of *S. aureus* RN4220 genome. (A) Whole-genome alignment of the parent strain NCTC8325 with RN4220 assemblies. The three regions indicated by the stars were present in NCTC8325 but not in the RN4220 genomes. The homologous regions were randomly colored for ease of distinction. The assembly GCA_000212435.2 appears longer due to a large number of contigs. The curation of three phages led to a modification of KMZ21_06995 C-terminus region (B), the promoter region of *yfkAB* (C), and the appearance of a new gene-*sph* (D). The RN4220 genome and phage removed from NCTC8325 are shown by yellow and black filled arrows, respectively. (E) Core proteome phylogenetic tree and analysis of gene gain/loss events in *S. aureus*. Numbers before the + and – sign indicated numbers of gene gain and loss events, respectively.

The complete genome sequence of *S. aureus* RN4220 was 2.7 Mb in length and harbored 2654 genes, including 19 rRNAs and 59 tRNAs (Table 2). We found that our assembly had 64 pseudogenes; the number did not differ significantly compared to previous assemblies, and these pseudogenes were not in proximity to prophages. To identify the genomic difference among the parent strain and RN4220 assemblies, we performed a whole-genome alignment of the parent strain NCTC8325 [24] (assembly accession: GCA_000013425.1). We found that large regions from NCTC8325 were deleted in the RN4220 genome (Figure 2A), which was also noted by a previous analysis using the draft genome [4]. We further analyzed the deleted region to find that these three regions included phages. The removal of phages in NCTC8325 resulted in modification of the C-terminus amino acid sequence of KMZ21_06995, the promoter region of *yfkAB*, and the appearance of new gene *sph* (Figure 2B – D). To gain an insight into the trend of gene gain and loss analysis, we used five other strains, including its parent and highly virulent strains. We found that among the examined strains, RN4220 had the lowest number of gene gains (Figure 2E).

**Table 2:** Analysis and comparison of general features of the current, complete *S. aureus* RN4220 genome with previous draft assemblies.

Features	Current assembly GCA_018732165.1	GCA_011751615.1	GCA_000212435.2 [4]
Assembly release date	2021/06/07	2020/03/25	2011/05/05
Sequencing technology	ONT minION Illumina HiSeq	BGIseq	Illumina GA II
Genome coverage	ONT minION: 23x Illumina HiSeq: 512x	372x	77x
Total Sequence length (bp)	2,697,195	2,657,542	2,663,395
No of contigs	1	27	118
Contig N50	-	174,720	80,460
Contig L50	-	4	13
Gene	2654	2626	2661
CDS	2572	2571	2604
Protein coding genes	2508	2517	2540
Pseudo Genes	64	54	63
RNA genes	78	-	-
Misc. binding	3	3	3
Misc. feature	3	3	3
ncRNA	3	3	3
Regulatory	10	10	10
rRNA	19	-	8
tmRNA	1	1	1
tRNA	59	51	45

### 3.2 Identification of fragmented genes and presence of repetitive elements

We compared our current assembly with the first genome assembly and found that many genes were fragmented and possibly not detected earlier. We found 39 new genes among the fragmented regions. Some notable genes identified included KMZ21_00310: *spa*; KMZ21_02625: *sdrC*; KMZ21_02630: *sdrD*; KMZ21_06705: *ebh*; and KMZ21_08630: *splF*. As these proteins are known to be involved in *S. aureus* pathogenesis through processes such as immune evasion, adhesion, complement resistance, and substrate acquisition, it is speculated that the virulence potential, based on the draft genome sequence, might have been overlooked. Similar results have been reported for relevant gene families comparing fragmented Illumina versus complete long read-based genome assemblies of the clinically highly relevant *Pseudomonas aeruginosa* [25]. Furthermore, 14 of these genes were located around the repetitive elements, suggesting the importance of long reads in the genome assembly (Table 3).

**Table 3:** Fragmented genes in the previous assembly [4], identified through the complete genome analysis. (-) in the position, the column indicates the gene in the complementary strand, and the + or - sign in the repeat column indicates the presence or absence of repetitive elements in the proximity of the gene, respectively.

SN	locus_tag	Length (AA)	product	position	repeat
1	KMZ21_00310	561	spa: Staphylococcal protein A	72912..74462(-)	-
2	KMZ21_00450	220	deoC: deoxyribose phosphate aldolase	103991..104653	-
3	KMZ21_00505	161	tnpA: IS200/IS605 family transposase	115089..115573	-
4	KMZ21_01125	117	transposase	263883..264233	-
5	KMZ21_01235	163	TIGR01741 family protein	288910..289401	+
6	KMZ21_01240	227	DUF5079 family protein	289609..290292	+
7	KMZ21_01265	165	TIGR01741 family protein	292558..293053	+
8	KMZ21_01275	227	DUF5079 family protein	293765..294448	+
9	KMZ21_01285	166	TIGR01741 family protein	295034..295534	+
10	KMZ21_01295	166	antitoxin YezG family	296056..296556	+
11	KMZ21_01300	166	TIGR01741 family protein	296567..297067	+
12	KMZ21_01855	518	restriction-modification system subunit M	403369..404925	-
13	KMZ21_02625	283	sdrC	559794..560643	-
14	KMZ21_02630	1383	sdrD	561010..565159	-
15	KMZ21_04460	461	mgtE	925408..926793	-
16	KMZ21_04700	36	hypothetical protein	972806..972916	-
17	KMZ21_05015	1150	pyruvate carboxylase	1037702..1041154	-
18	KMZ21_05785	567	proline tRNA ligase	1196448..1198151	-
19	KMZ21_06475	97	transposase	1341780..1342073	-
20	KMZ21_06560	160	transposase	1357791..1358271(-)	-
21	KMZ21_06705	9535	ebh: hyperosmolarity resistance protein	1384986..1413593(-)	-
22	KMZ21_06985	316	DUF	1469250..1470200(-)	+
23	KMZ21_07000	309	DUF	1473421..1474350(-)	+
24	KMZ21_07575	453	Acetyl-CoA carboxylase biotin carboxylase subunit	1578089..1579450(-)	-
25	KMZ21_08010	550	IS1182 transposase	1667723..1669374(-)	-
26	KMZ21_08245	505	hypothetical protein	1721716..1723233(-)	+
27	KMZ21_08370	220	transposase	1757495..1758156(-)	-
28	KMZ21_08480	541	IS1182 transposase	1775882..1777506	-
29	KMZ21_08525	333	menC	1785343..1786344(-)	-
30	KMZ21_08620	518	type I restriction-modification system subunit M	1802604..1804160(-)	+
31	KMZ21_08630	239	serine protease splF	1804523..1805242(-)	+
32	KMZ21_08640	35	hypothetical protein	1806211..1806318	+
33	KMZ21_08930	440	ISL3-like element IS1181 family transposase	1859410..1860729	-
34	KMZ21_09435	69	IS5/IS1182 family transposase	1936612..1936820(-)	-
35	KMZ21_10360	480	LmrS: multidrug efflux MFS transporter	2121681..2123123(-)	-
36	KMZ21_10440	541	IS1182 transposase	2140090..2141714	-
37	KMZ21_11355	185	transposase	2301164..2301720	-
38	KMZ21_12080	1499	E domain-containing protein	2445649..2450148(-)	+
39	KMZ21_12100	940	fibronectin-binding protein FnbB	2453135..2455957(-)	-

### 3.3 Complete genome facilitates the RNA-Seq analysis

**Figure 3:**
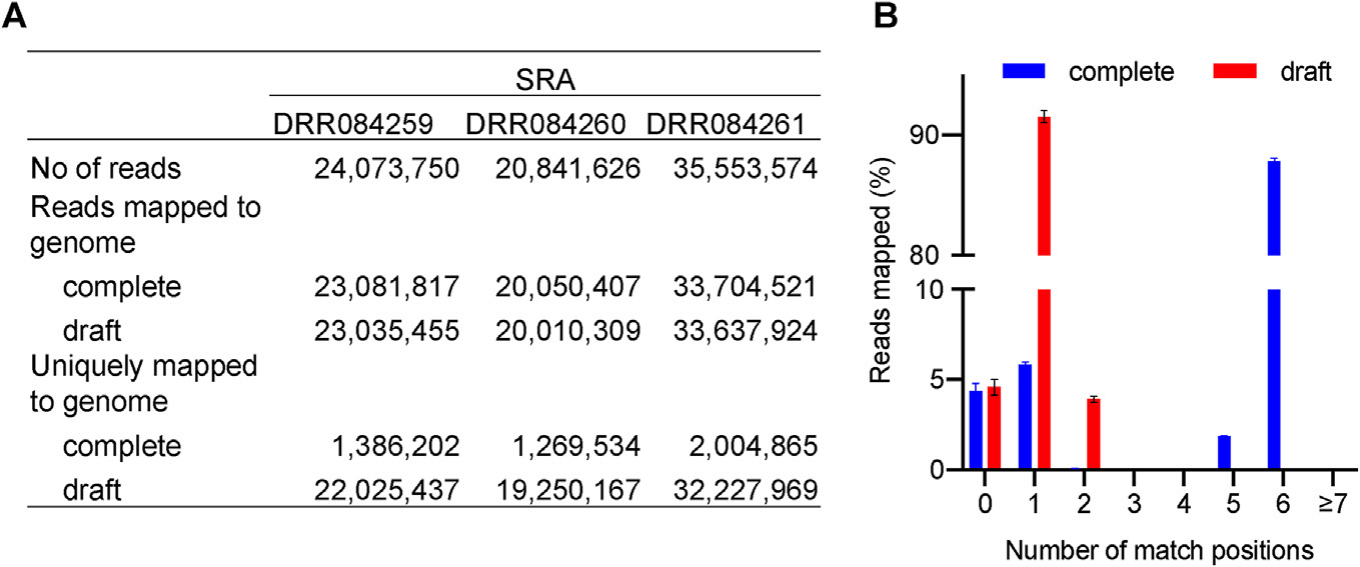
Comparison of complete (current) and draft [4] genome assemblies to analyze RNA-Seq results. Mapping (A) and match specificities (B) of RN4220 wild-type reads to the genome.

Using the publicly available data, we calculated the mapping of short RNA-Seq reads and compared the data with the complete and draft RN4220 genomes. We found that the number of reads mapped to the complete genome was slightly higher than that of the draft genome (Figure 3A). It is well known that the scaffolds in most draft genomes contain gaps [26] which may sometimes lead to a difference in the mapping of the reads and might be misleading while interpreting results. Therefore, we can expect that the increased mapping could be due to the mapping of the additional reads in the “gap” region that lies in between the contigs in the draft genome. Interestingly, the number of reads mapped specifically to the genome drastically reduced in the complete genome (Figure 3A). This could be because of the resolved repeat and duplicate regions in the complete genome, which appeared as a single contig in the draft genome. We found more than 80% of the reads were matched to six positions in the complete genome corresponding to the six copies of rRNA operons, which in the case of the draft genome was one (Figure 3B). Besides, the lack of the RN4220 complete genome required researchers to analyze RNA-Seq reads by mapping to the NCTC8325 genome [27, 28], where the results should be carefully interpreted, considering the differences among NCTC8325 and RN4220 genome sequences. In summary, these suggested the importance of a complete genome for omics-based analysis.

### 3.4 Downregulation of RN4220 genes involved in staphyloxanthin biosynthesis

**Figure 4:**
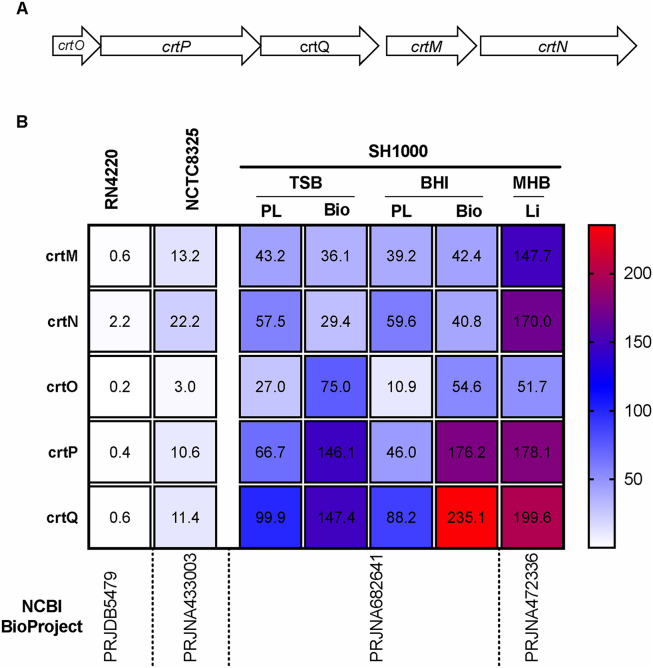
Staphyloxanthin biosynthetic gene cluster (A) and expression of the genes among NCTC8325, and SH1000, the *rsbU* repaired NCTC8325-4 strain (B). The short reads from the NCBI database (indicated Bioproject) were downloaded, mapped to respective genomes, and expression values, expressed as TPMs, were calculated using CLC Genomics Workbench. The mean of TPM values is shown. For RN4220, wild-type data was used. PL: Planktonic growth; Bio: Biofilm growth; Li: liquid culture.

*S. aureus* strains are usually distinguishable from other bacteria due to their yellow color, which is because of the production of yellow pigment staphyloxanthin. However, the RN4220 strain does not give the yellow pigmentation, producing very little or no staphyloxanthin. Staphyloxanthin biosynthetic genes are located in an operon *crtMNOPQ* (Figure 4A) [29,30] which is dependent upon the sigma factor B (SigB) [31]. SigB falls in an operon *rsbUVWSigB,* where RsbU and RsbV are the activators, and RsbW is the repressor of SigB [32, 33, 34]. It has been known that SigB is also controlled by YjbH [35] and CspA [36]. The parent strain of RN4220, NCTC8325, has a reduced ability to produce staphyloxanthin [37] which is attributed to a deletion of 11 bp in the *rsbU* gene. Since staphyloxanthin production is more pronouncedly decreased in RN4220 compared with NCTC8325 and NCTC8325-4 [37], we expected that the RN4220 strain might have some further alterations within these two operons. We aligned the amino acid sequences of the genes and found that these operons were conserved. Next, we aimed to examine the difference at the gene expression level. We looked for the raw RNA-Seq reads in NCBI SRA for NCTC8325, RN4220, and SH1000 strains. The *S. aureus* SH1000 [38] strain is a *rsbU*^+^ derivative of NCTC8325 and has the ability to produce staphyloxanthin. The reads were then mapped to complete genomes, and expression was analyzed using transcripts per million (TPM). We found that the *crt* operon was expressed at very low levels in RN4220, consistent with its pigment-less phenotype (Figure 4B). As we did not find genetic level changes within the *crt* operon, other regulatory factors are expected to play a role in the observed difference in staphyloxanthin production.

## 4 Conclusion

In this study, we completed the genome of a popular laboratory strain RN4220 for the first time. Consistent with previous reports [5, 8], we here provide an example of the importance of long reads in completing genomes containing repetitive elements, which is not possible by usual short-read sequences. The availability of the complete genome of this widely used strain is expected to serve as a platform for further genetic manipulation in a defined manner and robust omics-based analysis. In addition, we found that although the staphyloxanthin gene cluster was intact in RN4220, transcription of the operon was weak, resulting in a dramatic decrease in staphyloxanthin production and, hence, its pigment-less phenotype. Overall, the findings of this study provide valuable information on the *S. aureus* RN4220 strain by completing its genome, which will help interpret results in a defined manner by reducing biases and broadening our understanding of the genetic basis of various phenotypes.

## Declarations

### Author contribution statement

Suresh Panthee: Conceived and designed the experiments; Performed the experiments; Analyzed and interpreted the data; Wrote the paper.

Hiroshi Hamamoto: Conceived and designed the experiments; Performed the experiments; Analyzed and interpreted the data.

Atmika Paudel: Performed the experiments; Analyzed and interpreted the data; Wrote the paper.

Chikara Kaito, Yutaka Suzuki: Performed the experiments; Contributed reagents, materials, analysis tools or data.

Kazuhisa Sekimizu: Analyzed and interpreted the data; Contributed reagents, materials, analysis tools or data.

### Funding statement

Hiroshi Hamamoto was supported by 10.13039/501100001691Japan Society for the Promotion of Science (JSPS) KAKENHI [19K07140JP], Institute for Fermentation, Osaka (IFO) and ACRO Incubation Grants of Teikyo University. Suresh Panthee was supported by TBRF and IFO fellowships. Atmika Paudel was supported by 10.13039/501100001691JSPS KAKENHI 19K16653JP. Kazuhisa Sekimizu was supported by 10.13039/501100001691JSPS KAKENHI 21H02733 and 15H05783.

### Data availability statement

The complete genome of *S. aureus* RN4220 has been deposited to NCBI GenBank with accession CP076105.

### Declaration of interest's statement

The authors declare the following conflict of interests: K.S. is a consultant for Genome Pharmaceutical Institute Co., Ltd. The remaining authors declare no competing interests.

### Additional information

No additional information is available for this paper.
